# The Brain Health Index: Integrating Vulnerability, Resilience, and Functioning Into a Unified Measure of Cognitive Health and Neurodegenerative Risk

**DOI:** 10.1002/alz70860_105541

**Published:** 2025-12-23

**Authors:** Michael J Kleiman, Gregory Gibbs, James E. Galvin

**Affiliations:** ^1^ University of Miami Miller School of Medicine, Boca Raton, FL, USA

## Abstract

**Background:**

The Brain Health Index (BHI) is a comprehensive assessment tool that integrates three key components to create a unified measure of cognitive health: the Vulnerability Index (VI), which assesses both modifiable and non‐modifiable risk factors for cognitive impairment; the Resilience Index (RI), measuring six distinct modifiable factors known to strengthen resistance to impairment; and the Number‐Symbol Coding Task (NSCT), which evaluates executive functioning performance. This composite measure aims to provide physicians and researchers with a single score representing overall cognitive health and risk of future impairment. The BHI was developed to provide clinicians and researchers with a single, validated score representing overall brain and body health and enabling the tracking of aggregate risk factors over time.

**Method:**

The BHI utilized data from 265 participants (184 cognitively normal, 81 with mild cognitive impairment) enrolled in the University of Miami's Healthy Brain Initiative. Component weights were determined through factor analysis and SHAP feature importance based on random forest modeling, with manual correction. Comprehensive validation included correlational analyses with multiple health measures, including neuroimaging metrics, plasma biomarkers (amyloid beta, ptau217), gait parameters, and cognitive assessments.

**Result:**

The BHI demonstrated significant correlations with multiple health measures, including total cortical volume (*r* = ‐.279, *p* < .001), methylation clock derived phenotypic age (*r* = ‐.442, *p* < .001), MoCA score (*r* = .500, *p* < .001), and gait mean velocity (*r* = .480, *p* < .001). Higher BHI scores were associated with lower likelihood of cognitive impairment, confirmed through logistic regression (r2=‐.125, *p* < .001).

**Conclusion:**

The BHI successfully integrates measures of vulnerability, resilience, and cognitive performance into a unified metric that correlates significantly with established biomarkers of brain health and cognitive function. This comprehensive assessment tool provides clinicians and researchers with a validated measure for evaluating overall cognitive health and potential intervention needs. To further validate the BHI, a digital Brain Health Platform containing all components of the BHI has been deployed to 8,000 individuals throughout Florida.

